# Intelligent Tapping Machine: Tap Geometry Inspection

**DOI:** 10.3390/s23188005

**Published:** 2023-09-21

**Authors:** En-Yu Lin, Ju-Chin Chen, Jenn-Jier James Lien

**Affiliations:** 1 Department of Computer Science and Information Engineering, National Cheng Kung University, Tainan 701, Taiwan; p78061023@ncku.edu.tw; 2 Department of Computer Science and Information Engineering, National Kaohsiung University of Science and Technology, Kaohsiung 807, Taiwan

**Keywords:** tap, tapping, cutting angle, clearance angle, cone angles, length of the tool, tooth surface area, amount of thread removal

## Abstract

Currently, the majority of industrial metal processing involves the use of taps for cutting. However, existing tap machines require relocation to specialized inspection stations and only assess the condition of the cutting edges for defects. They do not evaluate the quality of the cutting angles and the amount of removed material. Machine vision, a key component of smart manufacturing, is commonly used for visual inspection. Taps are employed for processing various materials. Traditional tap replacement relies on the technician’s accumulated empirical experience to determine the service life of the tap. Therefore, we propose the use of visual inspection of the tap’s external features to determine whether replacement or regrinding is needed. We examined the bearing surface of the tap and utilized single images to identify the cutting angle, clearance angle, and cone angles. By inspecting the side of the tap, we calculated the wear of each cusp. This inspection process can facilitate the development of a tap life system, allowing for the estimation of the durability and wear of taps and nuts made of different materials. Statistical analysis can be employed to predict the lifespan of taps in production lines. Experimental error is 16 μm. Wear from tapping 60 times is equivalent to 8 s of electric grinding. We have introduced a parameter, thread removal quantity, which has not been proposed by anyone else.

## 1. Introduction

With the advent of smart manufacturing, real-time from machines in different locations can be accessed through cloud networks, allowing for integration and efficient management. Visual inspection systems, which replace traditional manual inspections, have been widely adopted to reduce reliance on human operators for monitoring machine operations. These systems enable rapid inspection of the external appearance of each product, including the geometric features of nuts and the wear of the tap. As metal products are predominantly processed using cutting tools, it is crucial to determine the lifespan of tooth taps and detect variations in their external features. This paper focuses on tooth tap inspection, aiming to produce Grade A nuts steadily and at a large scale. Grade A nuts are utilized in precision machinery sectors such as aerospace, naval vessels, power facilities, and buildings. Our collaborating manufacturer, whose skilled workers produce precision-Grade A nuts on the production line, relies on experience to replace taps. However, humans cannot maintain continuous focus on the production line for extended periods. With various instruments demanding attention on the production line, instances of oversight can occur. Therefore, machine vision inspection can help mitigate this issue. When the system detects taps that do not meet the specifications, it can provide an alert for tap replacement.

By employing computer vision instead of traditional measurement methods, tap geometry can be obtained quickly and accurately. To tap Grade A nuts at a large scale, it is necessary to calculate the cutting amount and wear of the tap during the tapping process. This involves predicting the location of the maximum tapping load, calculating tapers and taper lengths (with 5, 6, 7, and 8 peaks), nut thickness, and punched hole dimensions to determine whether they are too big or too small, among other parameters. The geometric design of the tap is measured before tapping, and the tapping process is recorded for hundreds of nuts. These recordings are then compared with the original information to establish standards for determining the amount of removed material and torque.

Currently, tap specifications include pitch, taper length, overall length, thread length, and shank diameter. However, these specifications do not account for wear-related parameters. Therefore, we propose the incorporation of clearance angle, cutting angle, tooth peak height, taper variation, taper angle, tooth area variation, and nut thread removal in the tap specifications. One of the challenges encountered in production is capturing metal shavings (noise) in the images. Therefore, the tap manufacturing team uses high-pressure cutting fluid during tapping to address this issue.

Taps are crucial tools in industrial machining, as most metal processing relies on their cutting capabilities. It is essential to quickly predict when the tap will produce non-compliant products. Due to the inability to inspect products on the production line individually, sampling is typically performed within a batch, which results in the possibility of mixing non-compliant products with conforming ones. By efficiently predicting or measuring the geometric condition of taps, manufacturers can reduce waste in producing products and save valuable time, ensuring the production of compliant products.

In the field of edge detection, a detailed introduction to prevailing edge detection methods has been provided [1]. The operational workflow of edge detection algorithms has been assessed [2]. A comparison was made between the edge detection methods of Prewitt and Canny [3]. Finally, a comparison was conducted among methods such as Roberts, Sobel, Prewitt, Laplacian, and Canny [4]. Under noisy conditions, the edge detection outcomes of Canny, Gaussian Laplacian, Roberts, Prewitt, and Sobel have been scrutinized [5]. Subsequently, the image edges were enhanced using a filtering technique, followed by the application of an enhanced ant colony optimization method for edge detection [6]. A unique approach has been employed for edge extraction, addressing the influence of random noise [7]. An adaptive median filter was used for Canny edge detection [8]. The Sobel edge detection results have been refined by applying the WNNM algorithm for denoising in noisy environments [9]. The regions of blurred edges have been eliminated [10]. The application of Canny for the detection of water ripples has been demonstrated [11]. For aerial coastline detection, a local adaptive Canny approach has been employed [12]. A three-threshold methodology has been adopted for Canny edge detection [13]. A novel variational model has been developed, which automatically and adaptively detects one or more predefined shapes from a given dictionary to guide the edge detection process [14]. The utilization of Mamdani (Type-2) fuzzy rules based on image gradient values for edge detection has been explored [15]. An enhanced Otsu method that incorporates edge detection and a decision tree classifier for crack identification has been proposed [16]. The Bi-Directional Cascade Network (BDCN) structure has been introduced [17], with individual layers supervised by specific labeled edges. The use of a spiking neural network for infrared edge detection has been presented [18]. A Deep Learning-based edge detector inspired by both HED (Holistically-Nested Edge Detection) and Xception networks was suggested [19]. For the paper that presented a new methodology for circle detection based upon randomized isosceles triangles sampling [20], the authors introduced the Curvature-Aided Hough Transform for Circle Detection (CACD) algorithm, which estimates the radius of circles based on curvature [21]. A novel multi-directional structural tensor has been constructed for corner detection, and a multi-scale corner measurement function is proposed to eliminate false candidate corners [22]. A purely event-based corner detector and a novel corner tracker have been introduced [23]. A new type of filter has been proposed, which is capable of simultaneously enhancing corners while suppressing edges and noise [24]. In addition, a multi-metric linear least squares iterative closest point algorithm for line detection in radar point clouds has been proposed [25]. Mu and Li (2018) proposed the Progressive Probabilistic Hough Transform (PPHT), which combined the Shi–Tomasi corner detection algorithm to enhance corner detection accuracy [26]. Even when we have identified a suitable edge detection method, there is still a chance of detecting defects in the tool handle area. Therefore, our challenge lies in finding a method to accurately fit a straight line even when there are defects at the edges, thereby eliminating or reducing the problem of overfitting in the least squares method [27]. Different least squares methods have been introduced [28]. Deep Learning has been utilized to predict road segments, and optimal curve fitting has been achieved through using the least squares method [29].

The study concludes the following: (1) Tapping tools change, especially in the cutting tooth peaks, as indicated by the cone angle variations during tapping operations. (2) Tapping tools have a clearance angle. (3) Measurement of cutting angles is only applicable to the detection of brand-new tapping tools. (4) Peak 5 and peak 6 experience the most significant wear. (5) Tooth peaks exhibit wear from peak 3 to peak 8. (6) Tooth peak areas decrease over time due to wear. (7) The amount to remove the thread increases as the tool becomes dull.

## 2. System Setup

**Figure 1:**
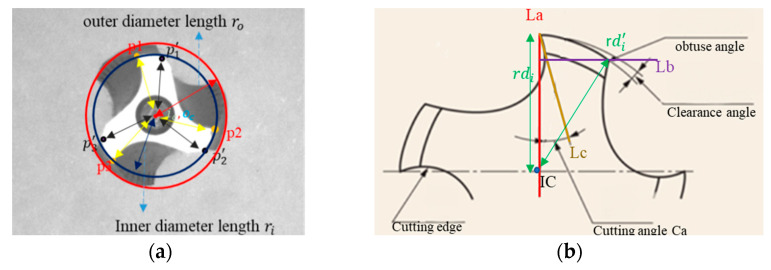
Tap surface detection target; (**a**) Tap placement inspection; (**b**) Tap cutting angle and clearance angle requirements.

**Figure 2:**
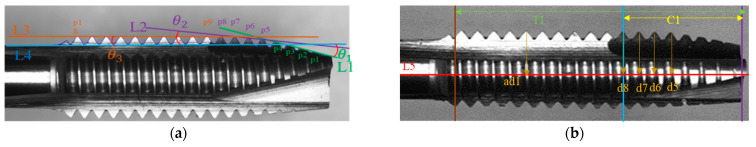
Tap side detection target; (**a**) Illustrations of the guide cone angle, cutting cone angle, and flute cone angle; (**b**) Illustrations of tooth peak height and tooth length.

**Figure 3:**
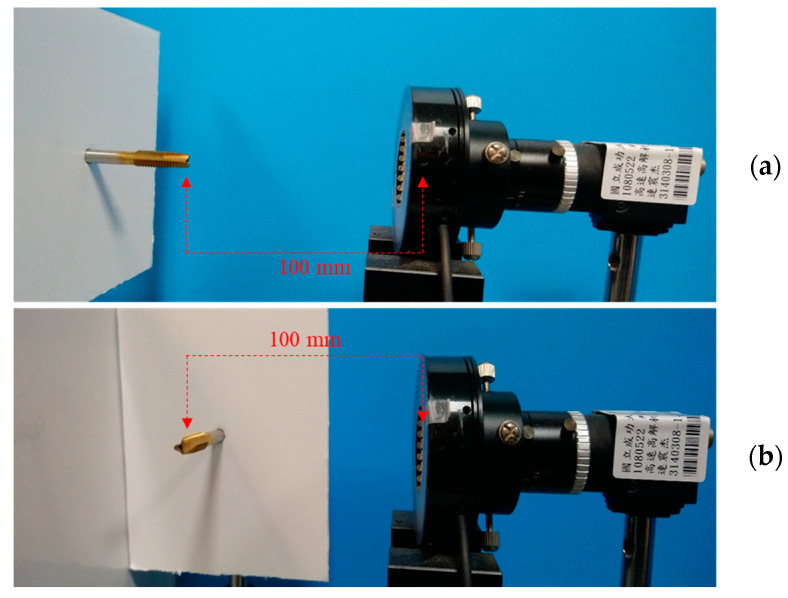
Experimental environment for tap inspection; (**a**) Frontal view; (**b**) Side view.

This paper proposes a three-part inspection system. Tap placement inspection, tap frontal inspection, and tap side inspection. Subsystem 1, as shown in Figure 1a, determines the outer $r_{o}$, inner $r_{i}$. Subsystem 2, as shown in Figure 1b, involves the identification of cutting angles ${ca}_{i}$, and clearance angles ${dr}_{o}$ and ${dr}_{i}$, and calculation of cone angle coa. Subsystem 3, depicted in Figure 2, focuses on measuring the guiding section angle $\theta_{1}$, cutting section angle $\theta_{2}$, polishing the threaded angle $\theta_{3}$, peak height $d_{i}$, cone length c1, tooth length t1, peak area $A_{i}$, and nut removal amount $\Delta A_{i}$. As illustrated in Figure 3, we captured the front and side images of the tap, simulating a floating working environment for the tap.

For the system setup, as shown in Figure 3, an industrial camera is used, and the tap is placed on a bracket to simulate the setup on the machine. The camera is positioned 100 mm away from the tap. The official tap inspection standards, as listed in Table 1, specify a pitch of 1.25 mm, guide section 5 mm, an overall length of 70 mm, and a shank diameter of 6.2 mm. However, these official standards only focus on specific parameters, such as pitch, overall length, and thread length without considering wear detection. Therefore, the official standards cannot be directly applied to tap inspection. A tap inspection concept based on brand-new taps is therefore proposed. When a tap fails to produce Grade A nuts, it would be replaced. Table 2 presents the proposed tap measurement criteria. As a result, we could not directly measure the geometric inspection results of the tap. Instead, we simulated wear through manual means and used precise Grade A nut standards with a tolerance of ±2% to eliminate taps.

The system is developed using Visual Studio 2012, featuring an MFC interface.

Table 1 contains the specifications provided by the tap manufacturer, while Table 2 represents the results obtained by our developed inspection system for newly manufactured taps. The 2% deviation corresponds to the A-Grade precision nut specification.

**Figure 4:**
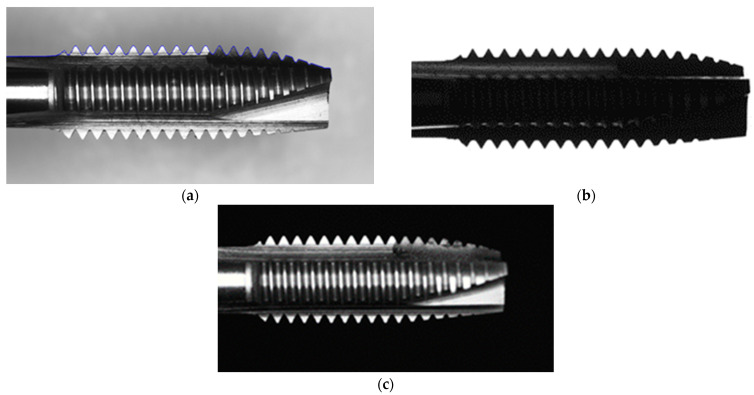
Three types of shooting environments for tapping machine inspection are as follows: (**a**) Using ring light with a background panel; (**b**) Using only a backlight panel; (**c**) Using only a ring light.

Three operating conditions were hypothesized: using a ring light with a background plate, using a backlight plate, and using a ring light combined with a background plate in Figure 4. We first captured the surface of the nut to verify the alignment of the tap. Then, we proceeded with the tap face inspection.

## 3. System Framework

**Figure 5:**
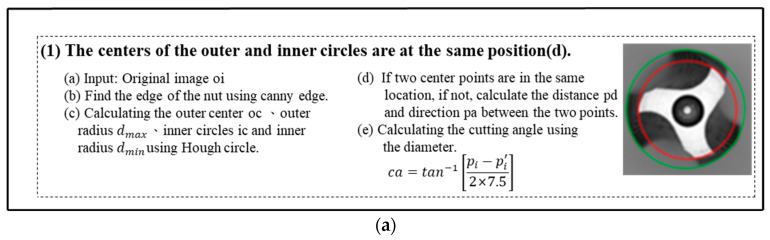

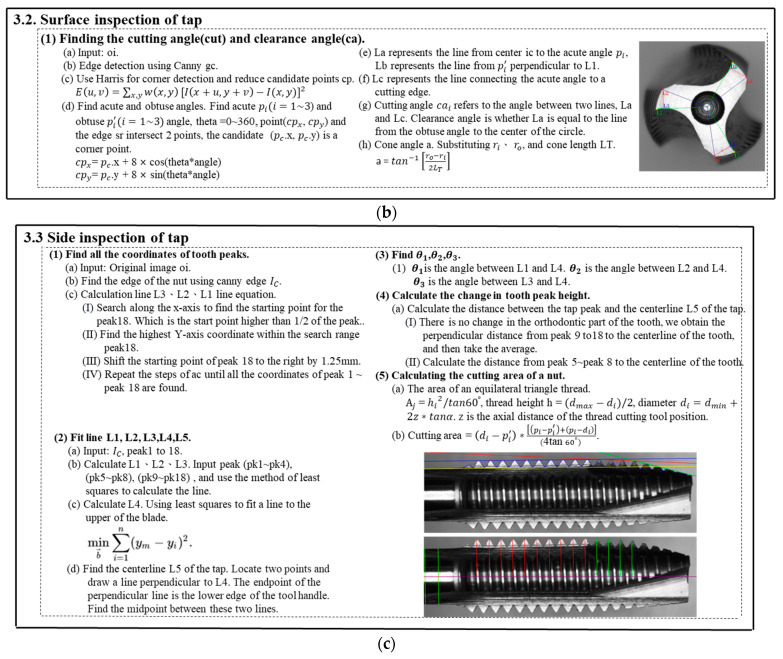
Flowchart of Tap Inspection; (**a**) Tap placement inspection; (**b**) Frontal inspection; (**c**) Side inspection.

This research divides the tap inspection system into three parts in Figure 5: tap placement, tap front-side inspection, and tap-side inspection. Tap placement is focused on determining whether the tap is properly positioned. Tap front-side inspection involves the detection of the cutting angle, clearance angle, and cone angle. Tap side inspection includes the measurement of guiding section angle, cutting section angle, polishing the threaded angle, cone length, blade length, tooth peak height variation, tooth peak area, and nut removal amount.

### 3.1. Tap Alignment Inspection

**Figure 6:**
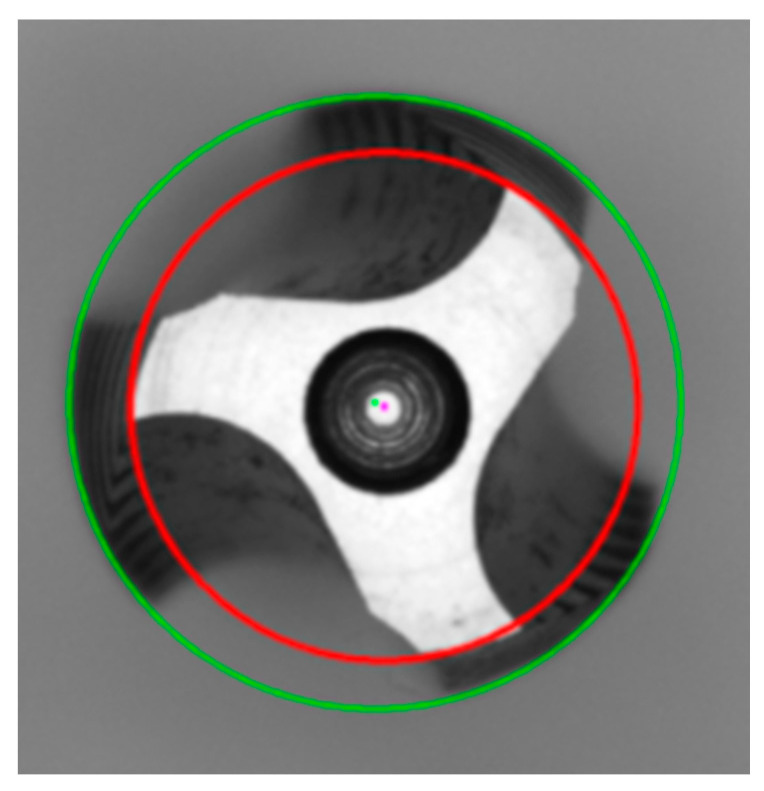
Inspection of tapping machine placement, Green represents the center to outer circle, and red represents the center to inner circle.

In order to detect whether the tap was correctly placed and to reduce errors in the inspection process, tap placement detection was performed prior to capturing the front side and side images of the tap. To facilitate subsequent concentricity inspection, the tap contour was extracted using the Canny edge detection algorithm. Then, the Hough circle transform was applied to detect the inner and outer circles of the tap. This method helps to determine the concentricity of the tap. If the distance between the circles exceeds a predefined threshold, it indicates an eccentricity issue with the tap, which requires adjustment. In this article, a standard radius of ±5 pixels was utilized to calculate the center and radius of the inner and outer circles on the front side of the tap in Figure 6. Once the tap placement is confirmed, images of the tap’s front side and side can be captured for further analysis.

(1) $$\left\{\begin{array}{c} General\;form:\;{\left(x-a\right)}^{2}+{\left(x-b\right)}^{2}=r^{2}.\\ Parametric\;form:\;x=a+rcos\theta ,\;y=b+rsin\theta \;(ln\;x-y\;domain).\\ Parametric\;form:\;a=x+rcos\theta ,\;y=b+rsin\theta \;(ln\;a-b\;domain). \end{array}\right.$$

During the tap detection process, we encountered two types of noise. The first type of noise was the iron chips and dust generated during the tapping operation; the second type was camera noise. To address the first type of noise, lubricating oil was used during tapping to flush and cool down the area, effectively removing iron chips and dust. As for the camera noise, the cone angle of the tap was then calculated based on the outer diameter and inner diameter.

(2) $$\alpha =\tan^{-1}\left[\frac{d_{max}-d_{min}}{2\times L_{T}}\right]=\tan^{-1}\left[\frac{7.729-6.58}{2\times 7.5}\right]=4.38^{{}^{\circ}}$$

$d_{max}$ refers to the outer diameter radius, $d_{min}$ refers to the inner diameter radius, and $L_{T}$ represents the length of the tap’s guiding portion.

Finally, the distance between the center of the inner circle $i_{c}$ and the center of the outer circle $o_{c}$ is used to determine whether the tooth position needs adjustment.

### 3.2. Surface Inspection of Tap

**Figure 7:**
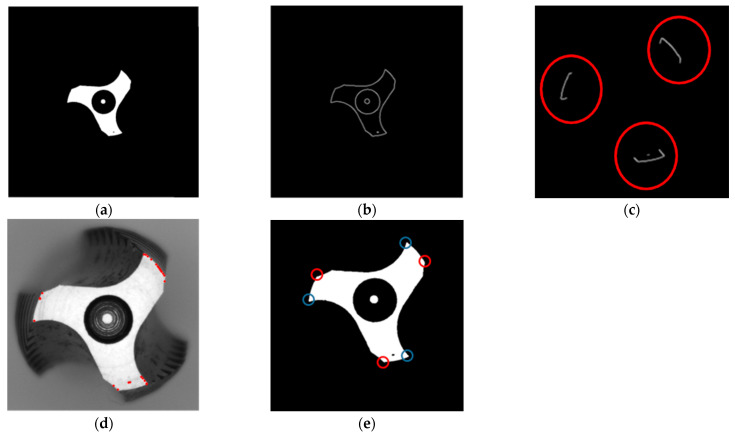
Identifying obtuse angles and acute angles; (**a**) Binary image; (**b**) Edge detection; (**c**) Preserve areas of interest; (**d**) Candidate points of acute and obtuse angles; (**e**) Identify the target points of acute and obtuse angles.

To determine the wear generated during tapping and whether the tap can produce Class A nuts, we propose to detect the cutting angle and clearance angle in the frontal tap inspection. By observing the wear on the front surface of the tap, we can assess if the tap can still be a Class A nut. Once the tap placement inspection is completed, the target contour needs to be extracted. Based on image observation, the region of interest appears close to grayscale 255 in Figure 7c, so we applied thresholding to extract the region of interest image. After extracting the tap contour, we used the Canny edge detection method to obtain the tap contour image. However, directly using the contour to identify the desired acute angles $p_{i}$, where *I* ranges from 1 to 3, and obtuse angles $p_{i}^{\prime }$, where *I* ranges from 1 to 3, is challenging. Directly applying corner detection would yield numerous corner points, making it difficult to select the required ones. Therefore, further refinement of the detection range is necessary. Since the tap specifications were fixed, we utilized the tap specifications to retain the edge information at the cutting edge. By taking the tap center as the reference point, we preserved the edge information within a radius of 180~200 pixels, obtaining an edge information image figure. Subsequently, the Harris algorithm is applied to the region of interest for corner detection.

(3) $$E\left(u,v\right)=\sum_{x,y}w\left(x,y\right)[I\left(x+u,y+v\right)-I(x,y){]}^{2}$$

In the equation, $w\left(x,y\right)$ represents the window at position (*x*, *y*), *I*(*x*, *y*) indicates the intensity at that position, and *I*(*x* + *u*, *y* + *v*) is the intensity at the moved window (*x* + *u*, *y* + *v*). The coordinates of the detected corner points are subsequently stored for further identification of acute and obtuse angles using a diagram in Figure 7d.

Next, we proceeded to identify three acute angles and three obtuse angles from the cutting edge. For each candidate point, which is represented as point[i] (${cp}_{x}$, ${cp}_{y}$), we performed the following evaluations: Taking the candidate point as the center, we drew a circle with a radius of 8 pixels. Condition one specified that the circle should intersect the tooth profile twice. Condition two stated that if the circle intersects the tooth profile twice, we could determine whether the angle formed is a straight line, an obtuse angle, or an acute angle using a diagram.

(4) $$\left\{\begin{array}{c} {cp}_{x}=p_{c}.x+8\times \cos(\mathrm{theta}\times \mathrm{angle})\\ {cp}_{y}=p_{c}.y+8\times \sin(\mathrm{theta}\times \mathrm{angle}) \end{array}\right.$$

Furthermore, we calculated the distance between the acute angles $p_{i}$, and the obtuse angles $p_{i}^{\prime }$, to determine if they lie on the same cutting edge. From the image, it can be observed that if the acute and obtuse angles are on the same cutting edge, they were with the shortest distance. This makes it easy to classify into three groups of acute angles and obtuse angles.

**Figure 8:**
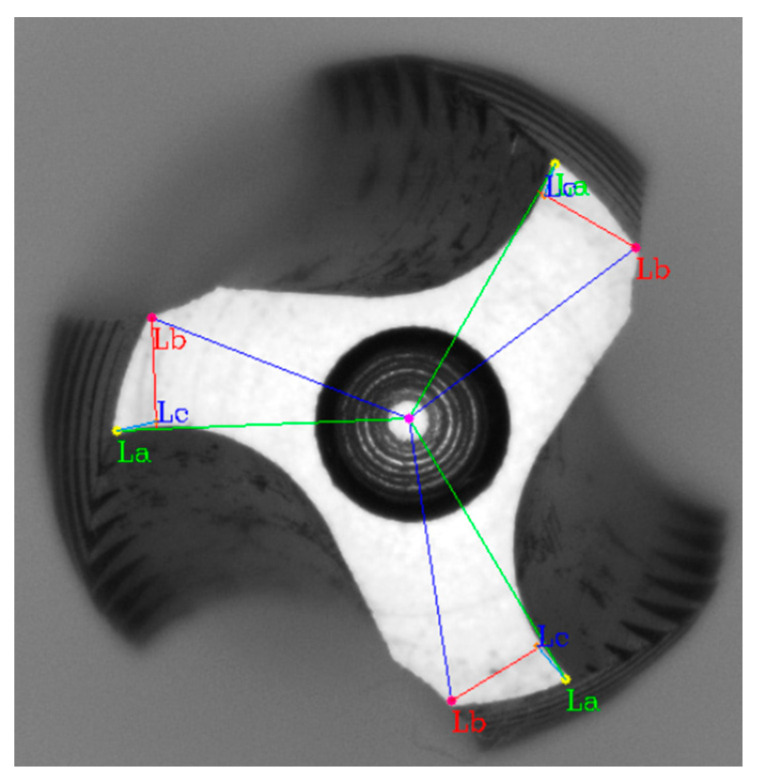
Tap cutting angle measurement results on the surface. The connection between the center and the acute angle is labeled as La, the perpendicular line drawn from the obtuse angle to La is labeled as Lb, and the intersection point between the acute angle and Lb, as well as the cutting edge, is labeled as Lc.

To determine the cutting angle ca, geometric relationships were applied. We connected the center of the inner circle ic, to the acute angle, forming line segment La. We drew line segment Lb perpendicular to La from the obtuse angle. Using the acute angle as the center, we further drew a circle with a radius of 5 pixels to find the coordinates that intersect the tooth profile. However, there would be two points of intersection. Line segment Lb was used to determine which point was closer to the intersection of La and Lb. The point closest to the intersection on the arc pta is the desired point. We drew line segment Lc from the acute angle through pta, reaching Lb. The angle between La and Lc is the cutting angle ca in Figure 8.

Next, we wanted to prove the existence of the clearance angle in the cutting edge. We calculated the distance $r_{i}$, between the acute angle $p_{i}$, and the center of the inner circle ic, as well as the distance $r_{o}$, between the obtuse angle $p_{i}^{\prime }$, and ic. By comparing $r_{i}$ and $r_{o}$. If the results of the distance are not equal, it might indicate an issue of clearance angle.

### 3.3. Tap Side Detection

The objective of the side face inspection was to determine the amount of material removed from the nut threads during tapping. To accomplish this, it was necessary to measure the tooth peak height on the side face of the tap. Furthermore, the tap cone angles of the guiding section $\theta_{1}$, cutting section $\theta_{2}$, and organize the threaded section $\theta_{3}$ were utilized to assess whether the tap is capable of producing A-Grade threads or not.

**Figure 9:**
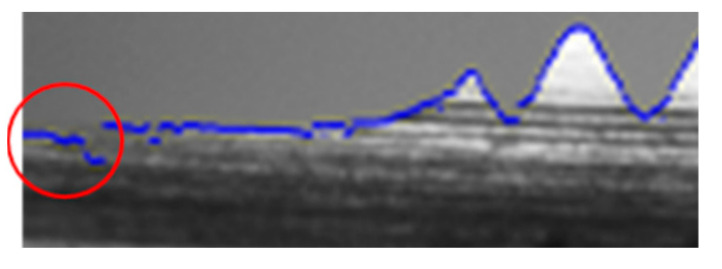
The grayscale value of the tool handle is too close to the background (red circle).

Once the tap placement inspection was completed in Section 3.1, side-face images of the tap were simultaneously captured. First, the tap contour needed to be extracted. In this paper, the Canny edge detection algorithm was used for edge detection. However, the edge detection process might be challenging because the tap shank might blend with the background in the image and the tap contour may not be accurately extracted in Figure 9. The straight characteristic of the tap shank can be utilized to weight the input coordinates.

To obtain accurate shank fitting results, we assigned the y-coordinate of the *x*-axis 0 to the x-coordinates from 0 to 9, the y-coordinate of the *x*-axis 10 to the x-coordinates from 10 to 19, and so on, until the *x*-axis reached 99.

#### 3.3.1. Calculate L1, L2, L3, L4, and L5

**Figure 10:**
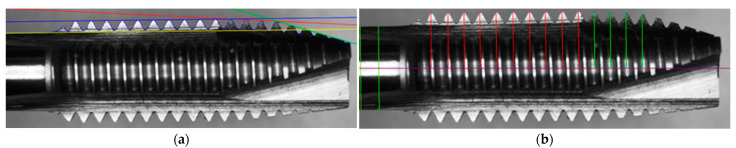
Side inspection results of the tap; (**a**) Detection of the guiding section angle, cutting section angle, and form section angle; (**b**) Tooth peak height inspection.

The objective was to identify the lines L1, L2, L3, L4, and L5 in Figure 10. First, we located the tooth valley of peak 18. For this purpose, we used half of the tooth peak height as the initial point when searching for the tooth valley. The reason for choosing half as the initiating point is that there was a small tooth peak to the left of peak 18 in the image Figure 9, and its height did not exceed half of peak 18 height. We found the starting point peaks18 of tooth peak 18 from the tap contour. We proceeded to locate the tooth valley peakv18 of peak 18. Using peaks18 as the reference point, we could then search for the highest y-value within the range of peaks18.x − 40 to peaks18.x.

Next, we conducted a search for peak 18 by identifying the coordinate with the smallest y-value within the search range of peakv18.x to peakv18.x + 80. We chose a range of 80 pixels because the tooth peak width is 80 pixels (≈1.25 mm). Next, search for peak 17. The first search result is peak 17 valley peakv17, here offset 1.25 mm to the right from peakv18, obtaining the starting point for peak 17 valley search, denoted as peak17s. Using the maximum y-value in the region, we located the coordinate peak17v within the search range of peaks 17.x − 15 to peaks 17.x + 15. We repeated the above process to determine all the peaks from peak 18 to peak 1.

Next, we stored the contour coordinates of the handle required for calculating line segment L4. We stored the first 100 coordinates here.

We calculated the cone angles $\theta_{1}$, $\theta_{2}$, and $\theta_{3}$ for the guiding section, cutting section, and polishing the threaded section, respectively. We input coordinates and then employed the least squares method to fit a line segment L1 to L4 into the form *y* = *k* × *x* + *b*:

(5) $$k=\frac{\sum xy-n\bar{x}\bar{y}}{\sum x^{2}-n{\bar{x}}^{2}}$$

To obtain the slope k and solve the equation for obtaining b. L1 represents input from peak 1 to peak 4, L2 represents input from peak 5 to peak 8, and L3 represents input from peak 9 to peak 18. Next, the upper contour of the handle is fitted to generate the line segment L4 using the tool handle coordinates. To minimize the impact of shadows and reflections in Figure 10. Line segment L4 is then fitted using the method of weighted least squares estimation. This approach minimizes the influence of reflections and shadows.

(6) $$\mathrm{Minimize}\;\Sigma (w_{i}\;\times \;{(y_{i}-(a\;*\;x_{i}\;+\;b))}^{2})$$

$w_{i}$ is the weight of the *i*-th data point. For the first 10 coordinates, $w_{i}$ = 1.0, for the next 10 coordinates, $w_{i}$ = 2.0, and for the remaining data points, $w_{i}$ = 1.0 (or other weight values can be set as needed). Variables a and b are the fitting parameters; they are the slope and intercept of the linear fitting function.

To locate the centerline L5 of the tap, we used the 10th and 100th coordinates stored in the tap contour, denoted as L5p1 and L5p2, respectively. We drew two perpendicular lines, m1 and m2, to line L4 using these coordinates. We recorded the coordinates where m1 and m2 intersect with the lower-end contour of the tap, denoted as L5p$1^{\prime }$ and L5p$2^{\prime }$. We calculated the midpoint coordinate m10 between L5p1 and L5p$1^{\prime }$. Similarly, we calculated the midpoint coordinate L5mp2 between L5p2 and L5p$2^{\prime }$. Finally, we could identify the centerline L5 of the tap by drawing a line through points L5mp1 and L5mp2.

#### 3.3.2. Calculate $\theta_{1}$, $\theta_{2}$, $\theta_{3}$

Calculate the angle between L1 and L4 as the guiding angle $\theta_{1}$, the angle between L2 and L4 as the cutting angle $\theta_{2}$, and the angle between L3 and L4 as the polishing the threaded angle $\theta_{3}$.

#### 3.3.3. Calculate the Tooth Peak Height

The variation in height was calculated after wear on the tooth peaks, the length of the tooth cone section was measured to evaluate if it is affected by the wear, and the amount of material removed from the nut was calculated. The distances $d_{i}$ can be calculated from each peak (peak 18 to peak 5) to the line L5. The average value, ad1, of $d_{18}$ to $d_{9}$ shall be calculated as well. Finally, individual tooth peak heights shall be recorded for peak5 to peak 8.

To calculate the length of the tap and the length of the cone section, first locate the mapping position of the tooth valley peakv18, at the trailing edge of the blade on the tap centerline L5, denoted as bf. Identify the mapping position of the tooth valley peakv8, at the end of the tap cone section on the tap centerline L5, denoted as cf. Find the position of the tap crest face *sp*, by starting from the peak1.x position and searching for the contour’s vertical fracture position. Determine if the consecutive coordinates have a difference greater than a threshold value. The point sf maps to the position sp on the tap centerline, L5. Calculate the positions bf, cf, and sp on the line L5 using the following formula:

(7) $$H=P+t\overset{⃑}{n},t\in R$$

*H* represents the mapping of point *P*(p.x, p.y) on the line ax + by + c = z. *H* = (p.x + at, p.y + bt), where *H* is a point that satisfies the equation ax + by + c = z. The length of the tap is the distance between bf and sp. The length of the cone section is the distance between cf and sp.

**Figure 11:**
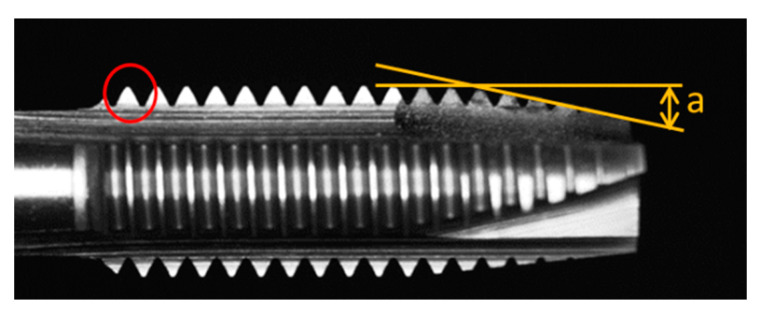
Area measurement of the tooth edge, the orange represents the cone angle when viewed from the front.

To calculate the amount of material removed from the nut thread in Figure 11, we can use the previously obtained tooth peak height $d_{i}$, shank width $d_{o}$, and tooth valley positions ${peakv}_{i}$. First, we had to calculate the area of the tooth peak’s equilateral triangle in its original state:

(8) $$A_{max}=a\times \frac{h}{2}=\frac{1}{2}\times \left(\frac{2h}{{\tan60}^{{}^{\circ}}}\right)\times h=h^{2}/{\tan60}^{{}^{\circ}}$$

a is the cone angle, and the tooth height is given by *h* = $(d_{max}-d_{o})/2$, where $d_{max}$ is the distance from the tooth peak to the furthest point on the tooth from the center. Using this formula, we can calculate the equilateral triangle area for a brand-new tooth peak (shown in red circle). Next, we calculate the triangle area for the tooth peak with wear.

(9) $$A_{i}=a_{i}\times \frac{h_{i}}{2}=\frac{1}{2}\times \left(\frac{2h_{i}}{{\tan60}^{{}^{\circ}}}\right)\times h_{i}={h_{i}}^{2}/{\tan60}^{{}^{\circ}}$$

$a_{i}$ represents the cone angle, where the tooth height is given by *h* = $({peak}_{max}-d_{i})/2$. The corresponding diameter is $d_{i}={peakv}_{min}+2z\times tan\alpha$, where z is the axial distance from the tooth peak. ${Peak}_{max}$ is the distance from the highest tooth peak to the center of the tooth, and ${peakv}_{min}$ is the distance from the lowest tooth valley to the center of the tooth. Finally, we calculate the amount of material removed from the nut (cutting area).

(10) $$\Delta A_{i}=A_{max}-A_{i}=(h^{2}-h_{i}^{2})/\tan60^{{}^{\circ}}$$

(11) $$=[({peak}_{max}-{peaks}_{min}{)}^{2}-({peak}_{max}-d_{i}{)}^{2}]/(4\tan60^{{}^{\circ}})$$

(12) $$=\left(d_{i}-{peaks}_{min}\right)\times \frac{\left[\left({peak}_{max}-{peaks}_{min}\right)+\left(peal-d_{i}\right)\right]}{\left(4\tan60^{{}^{\circ}}\right)}$$

$\Delta A_{i}$ represents the amount of material removed from the nut, $A_{max}$ is the area of the intact triangular tooth peak, and ${\;A}_{i}$ is the area of the specified worn tooth peak. The height *h* is given by $({peak}_{max}-d_{i})/2$, where $h_{i}$ is the specified tooth peak height. ${Peak}_{max}$ is the distance from the highest tooth peak to the center of the tooth, and ${peak}_{min}$ is the distance from the lowest tooth valley to the center of the tooth. By comparing the changes in tooth peak area and the amount of material removed from the nut, we can determine if the nut meets the requirements for an A-Grade thread after tooth tapping.

## 4. Experimental Results

**Figure 12:**
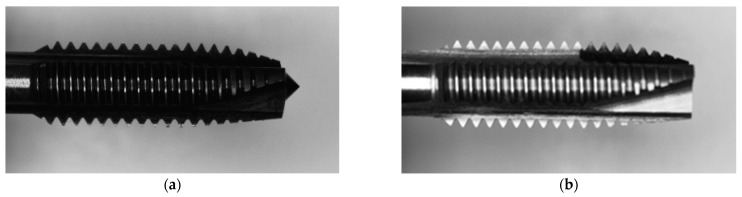
Two different materials of tapping tools: (**a**) Stainless steel; (**b**) Titanium alloy.

This research is a subproject of smart manufacturing for intelligent tapping machines responsible for simulating and developing geometric inspection techniques for nuts and tapping tools. Practical inspection techniques are developed based on the inspection requirements proposed by the tapping machine manufacturing team. Two types of tapping tools were utilized, both with the same external design but different materials: one made of titanium and the other made of stainless steel. Figure 12a shows the stainless-steel tap of model 6912, and Figure 12b shows the titanium tap of model 7912p. We conducted experiments on three sets of tapping tools, including brand-new tapping tools, tapping tools used on 60 nuts, and artificially polished tapping tools. For the artificially polished tapping tools, we captured images starting from the brand-new state, recorded the duration of electric grinding after fixing and captured images after each grinding to simulate the gradual wear of tooth edges. In this study, we measured the geometric characteristics of the tapping tools with two different materials and our proposed inspection method. Based on the inspection results, the changes in the tooth edge area and the amount of thread removal from the nuts were calculated.

Industrial cameras were used to capture images of the frontal and side views of the tapping tools. The original size of the camera was 4000 × 3000 pixels, but we selected a cropped image size of 2048 × 1024 pixels based on the distance, focal length, and size of the tapping tool in the frame. According to calculations, each pixel corresponds to a size of 16 μm.

Since the tapping tools in the tapping machine manufacturing team might be floating, it is necessary to ensure their proper placement before conducting geometric inspections and operations. Once the tools were properly placed, the geometric inspection of the frontal and side views of the tapping tools would be carried out. Two cameras would be installed on the production line specifically for capturing the frontal and side views of the tapping tools.

We can extract the cutting angle, clearance angle, and cone angle calculations from the frontal view of the tapping. From the side view of the tapping tool, the angles of the guiding section, cutting section, and thread polishing section were inspected, and the wear of each tooth peak were measured. Inspections were also conducted on the blade length and taper length. Finally, we calculated the tooth peak area and the variation in thread-cutting volume for the nut.

Table 3 provides an explanation of the taps we have tested. “New” represents brand-new taps that have never been used. “Tapping 60 times” refers to taps used to tap 60 nuts on the tapping machine. “Artificial gradual wear” taps are obtained by initially capturing images of brand-new taps and then subjecting them to artificial wear.

**Figure 13:**
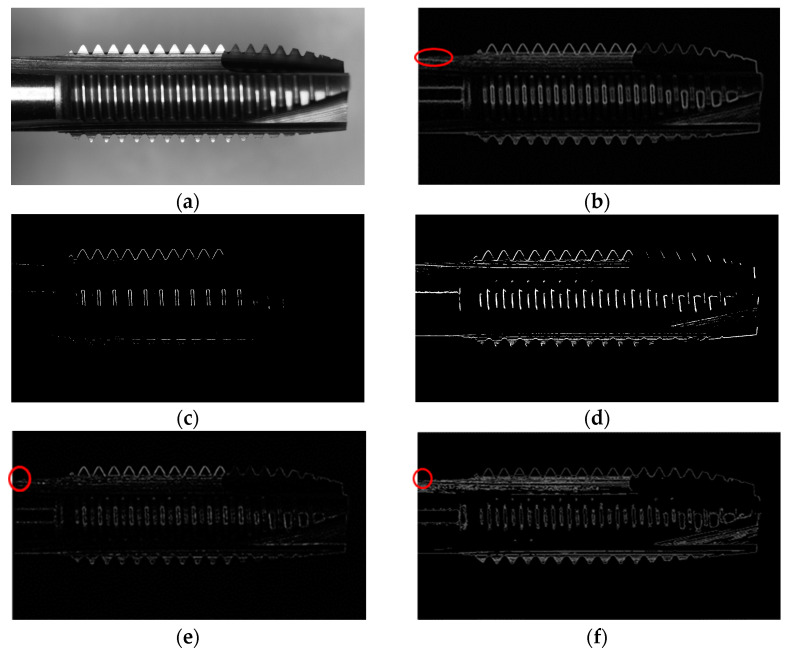
Results of different edge detection methods for tap detection, the red represents the missing portion: (**a**) Original image; (**b**) Sobel; (**c**) Roberts; (**d**) Prewitt; (**e**) Dual Parity Morphological Gradients; (**f**) Canny.

From Figure 13, we can observe the results of different edge detection methods. In Figure 13b, the Sobel operator failed to distinguish the handle from the background due to their similar intensities. In Figure 13c, the Roberts operator is not suitable for toothpick detection as it could not accurately detect the edges in the handle region and some peaks. In Figure 13d, the Prewitt operator performs better than Roberts, but there were still areas in the handle and toothpick regions that could not be detected. In Figure 13e, the Dual Parity Morphological Gradients method still had missing regions in the handle area. Figure 13f shows that the Canny operator, with its modified settings, could accurately detect the straight handle line L4.

**Figure 14:**
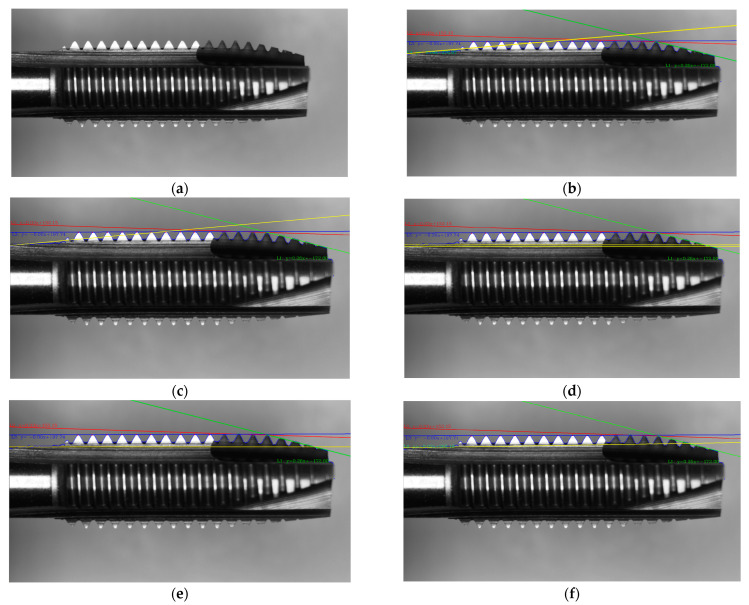
Detecting the results of the L4 baseline using different linear fitting methods. (**a**) Original; (**b**) Least squares; (**c**) Total least squares; (**d**) Hough line; (**e**) Ransac; (**f**) Weighted least squares.

In Figure 14, different methods were used to fit a straight line to the blurry image of a knife handle. Figure 14a shows the original image. Figure 14b represents the least squares method, but it can be observed that the original least squares method does not handle the edges with colors close to the background well. Figure 14c shows the total least qquares method, which also fails to address the issue. In Figure 14d, the Hough line method is used, but failed to both recognize a single straight line and identify additional erroneous lines. Changing the parameters only results in either the inability to recognize the line or detecting two lines instead of one. Figure 14e depicts the Ransac method, which can handle cases where the knife handle’s color is close to the background. Lastly, Figure 14f presents the improved least squares method with a modified input, resulting in a fitted straight line that closely aligns with the knife handle’s edge.

Table 4, Table 5 and Table 6 present the geometric inspection results for the tapping tools after 60 tapping compared to the brand-new tools. The table is divided into measurements for the helix angle, tooth peak height, and tooth peak area. The tooth peak height was found to decrease compared to the brand-new tapping tools, indicating wear. The tooth peak area also decreased due to wear, and the amount of material that needed to be removed increased as the tool became dull.

**Figure 15:**
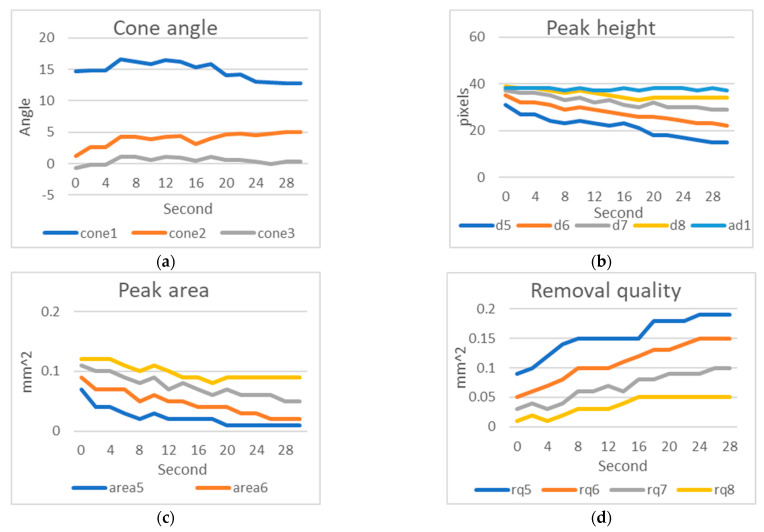
Results of artificial wear on stainless steel 6912: (**a**) Cone angle; (**b**) Tooth peak height; (**c**) Tooth peak area; (**d**) Amount of thread removal from the nut.

Based on the simulated wear detection results for stainless steel 6912, according to actual measurements, peak 3 and peak 4 exhibit wear, while the first two teeth did not come into contact with the inner wall of the nut. The cone angle $\theta_{1}$ decreased due to the wear of peak 3 and peak 4. The distances d5 to d7 gradually became shorter over time, while d8 slightly decreased and then stabilized in condition. The average distance ad1 in the overall tooth section remained unchanged, with an average value ranging from 37 to 38 pixels Figure 15.

The tooth peak areas from peak 5 to peak 8 decreased gradually due to wear, leading to an increase in the thread removal amount. However, the removal amount for rq8, tabilized after an initial increase.

**Figure 16:**
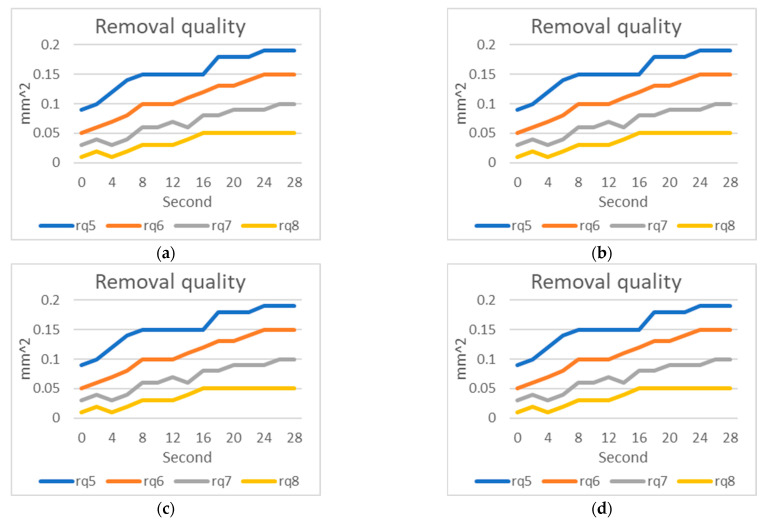
Results of artificial wear on titanium 7912p: (**a**) Cone angle; (**b**) Tooth peak height; (**c**) Tooth peak area; (**d**) Amount of thread removal from the nut.

First, we calculated the precision of our inspection system. After capturing an image, we adjusted the background panel brightness after a certain time and captured another image. We observed that the tooth peak height occasionally had a 1-pixel discrepancy. Knowing that 1 pixel on our industrial camera represents 16 μm, we determined that our inspection system has a maximum error of 16 μm. In our manual wear experiments using electric grinding on stainless steel taps (6912 cycles), we found a 1-pixel discrepancy in the experimental results. Taking the example of peak 5 height in Figure 15b, we observed that the inspection results fell within a range of 16 to 17 pixels, which matched our expectations. Additionally, from Table 5 and Figure 15b, we learned that the wear from tapping 60 times is equivalent to 8 s of electric grinding. In Figure 15a and Figure 16a, the change in cone angle 1 is becoming flatter and actual tapping causing peak 3 and peak 4 to touch the nut’s internal hole and wear matched. The change in cone angle 2 becoming inclined also confirmed that peak 5 in the cutting section experiences the most significant wear, aligning with the results in Table 5. The slope matching real wear was further supported by observing the tooth peak height variation in Figure 16b. Comparing it to the results in Table 5 for tapping 60 times, we found that peak 5 had worn by 7 pixels, which is equivalent to tapping 60 times or 8 s of grinding. Peak 6 had reduced in height by 6 pixels, and peak 7 had reduced by 4 pixels during 8 **s** of electric grinding. These results closely resembled the electric grinding outcomes, indicating that our simulated wear results are close to actual wear. Lastly, the tooth peak height after electric grinding matched normal tapping wear. In Figure 15c, tooth peak areas of 0.09, 0.12, and 0.15 closely matched the real tapping results of 0.09, 0.13, and 0.14. These results led us to conclude that our simulated wear results closely mimic normal tapping wear.

## 5. Conclusions

This study aims to produce Grade A precision nuts with tapping tools. To achieve this, it is important to determine the time when several nut-tapping operations started to produce a significant number of non-precision Grade A nuts. The proposed inspection system aims to be installed on the machine, ensuring fast and accurate detection. The system has several advantages: firstly, it eliminates the need for additional inspection equipment. Secondly, it directly integrates the camera into the production line. Thirdly, it offers three predefined inspection environments to accommodate different installation conditions: using a ring light with a background plate, using a ring light without a background plate, or using only a backlight panel. Lastly, the system provides an early indication to replace worn tapping tools when they can no longer produce Grade A nuts.

The proposed method focuses on the detection of worn tapping tools. The inspection is categorized into front-face and side-face measurements. The front-face inspection involves implementing Hough circle detection to verify the placement of the tapping tool, measuring the cutting angle based on geometric variations, and assessing the presence of backlash angle to extend tool life. The side-face inspection identifies tooth peaks and employs the least squares method to measure the lead angle of the guide section, cutting section, and overall tooth section. To address the issue of the handle having a similar color to the background, we introduce weighted input coordinates to obtain a more accurate fitting of the handle’s straight line. The tooth peak heights and tapping tool length are measured based on the centerline. The changes in tooth peak area and thread removal should also be calculated. We considered how to assist in locating the tap’s imaging position, confirmed the inspection error to be within 16 μm through repeated imaging, and introduced a weighted linear detection method to address issues related to taps having similar grayscale values to the background and reflections.

In the future, the utilization of Deep Learning in conjunction with camera replacement has potential to significantly enhance detection accuracy. Deep Learning can be employed to incorporate edge detection, circle detection and line detection. Research into these methods can provide insights into their performance improvement relative to conventional approaches.

## Figures and Tables

**Table 1:** Official tap standards.

Official Tap Standards	
Pitch P	1.25 mm ± 2%
Guide section Lc	5 mm ± 2%
Overall length L	70 mm ± 2%
Length of thread engagement T1	22 mm ± 2%
Shank diameter	6.2 mm ± 2%

**Table 2:** Our tap standards.

Our Tap Standards		
Inside radius $r_{i}$	Edge image	6.58 mm ± 2%
Outside radius $r_{o}$	Edge image	7.70 mm ± 2%
Cutting angle ${ca}_{i}$	Lc & Cutting edge	4.38° ± 2%
Guide cone angle $\theta_{1}$	L1 & L4	14.40° ± 2%
Cutting cone angle $\theta_{2}$	L2 & L4	1.10° ± 2%
Flute cone angle $\theta_{3}$	L3 & L4	0° ± 2%
Peak height $d_{i}$	${Peak}_{i}\;$ & L5	0.63 mm ± 2%
Total length $t_{l}$	Peakv18 & L5	24 mm ± 2%
Cone length $c_{l}$	Peakv8 & L5	11 mm ± 2%
Peak area $a_{i}$	$d_{i}$	0.19 mm^2^ ± 2%
Thread Removal Amount ΔA	$A_{max}$ & $a_{i}$	0.08 mm^2^ ± 2%

**Table 3:** Detecting the number of tapping tools.

	New	Tapping 60 Time	Artificial Gradual Wear
6912	1	0	6
7912p	2	2	6

**Table 4:** The Cone angle between brand-new titanium material tap and those used for tapping 60 times.

	$\theta_{1}$	$\theta_{2}$	$\theta_{3}$
New	$14.40^{{}^{\circ}}$	$1.11^{{}^{\circ}}$	$0.04^{{}^{\circ}}$
60 times	$13.79^{{}^{\circ}}$	$1.52^{{}^{\circ}}$	$0.16^{{}^{\circ}}$

**Table 5:** Tooth peak height comparison between brand-new titanium material tap and those used for tapping 60 times.

	d5	d6	d7	d8	ad1
New	41 pixel	46 pixel	45 pixel	46 pixel	43 pixel
60 times	34 pixel	40 pixel	41 pixel	40 pixel	40 pixel

**Table 6:** Tooth peak area and amount of thread removal comparison between brand-new titanium material tap and those used for tapping 60 times.

	area5	area6	area7	area8	rq5	rq6	rq7	rq8
New	0.14 mm^2^	0.18 mm^2^	0.19 mm^2^	0.18 mm^2^	0.04 mm^2^	0.03 mm^2^	0.04 mm^2^	0.08 mm^2^
60 times	0.09 mm^2^	0.13 mm^2^	0.14 mm^2^	0.13 mm^2^	0.09 mm^2^	0.08 mm^2^	0.09 mm^2^	0.14 mm^2^

## Data Availability

The data are not publicly available due to confidential component production by our collaborating partner.
